# Extracellular Alpha-Synuclein: Mechanisms for Glial Cell Internalization and Activation

**DOI:** 10.3390/biom12050655

**Published:** 2022-04-30

**Authors:** Cecilia Chavarría, Rodrigo Ivagnes, José M. Souza

**Affiliations:** Departamento de Bioquímica, Centro de Investigaciones Biomédicas (CEINBIO), Facultad de Medicina, Universidad de la República, Av. Gral. Flores 2125, 11400 Montevideo, Uruguay; cchavarria@fmed.edu.uy (C.C.); rivagnes@fmed.edu.uy (R.I.)

**Keywords:** extracellular alpha-synuclein, oligomers, fibrils, astrocytes, oligodendrocytes, microglia

## Abstract

Alpha-synuclein (α-syn) is a small protein composed of 140 amino acids and belongs to the group of intrinsically disordered proteins. It is a soluble protein that is highly expressed in neurons and expressed at low levels in glial cells. The monomeric protein aggregation process induces the formation of oligomeric intermediates and proceeds towards fibrillar species. These α-syn conformational species have been detected in the extracellular space and mediate consequences on surrounding neurons and glial cells. In particular, higher-ordered α-syn aggregates are involved in microglial and oligodendrocyte activation, as well as in the induction of astrogliosis. These phenomena lead to mitochondrial dysfunction, reactive oxygen and nitrogen species formation, and the induction of an inflammatory response, associated with neuronal cell death. Several receptors participate in cell activation and/or in the uptake of α-syn, which can vary depending on the α-syn aggregated state and cell types. The receptors involved in this process are of outstanding relevance because they may constitute potential therapeutic targets for the treatment of PD and related synucleinopathies. This review article focuses on the mechanism associated with extracellular α-syn uptake in glial cells and the consequent glial cell activation that contributes to the neuronal death associated with synucleinopathies.

## 1. Introduction

### 1.1. Synucleinopathies

“Involuntary tremulous motion, with lessened muscular power, in parts not in action and even when supported; with a propensity to bend the trunk forward, and to pass from a walking to a running pace, the senses and intellects being uninjured” [1].

Parkinson’s disease (PD) is an extrapyramidal syndrome clinically characterized, as described by James Parkinson in 1817, by rigidity, tremor, and bradykinesia [1,2]. The progressive loss of dopaminergic neurons in the substantia nigra and the presence of alpha-synuclein (α-syn) protein inclusions in neuronal cell bodies, known as Lewy bodies (LBs), are the pathological hallmarks of PD [3,4,5]. The underlying pathological basis of PD remains unclear. α-Syn is the main fibrillar component of LBs in sporadic and inherited PD, and it is also part of a group of neurological diseases known as synucleinopathies, which includes multiple system atrophy (MSA), pure autonomic failure, and dementia with LBs [4,5,6,7]. It has been demonstrated that duplications and triplications of *SNCA* (genetic locus that encodes α-syn) are related to accelerated disease onset and progression [2,5,8,9], suggesting that increased α-syn expression is sufficient to trigger the disease process. Many animal models overexpressing wild-type or mutant forms of α-syn show cytoplasmic inclusions and motor deficits [2,5,10,11,12,13,14,15].

### 1.2. Alpha-Synuclein and Aggregation Process

α-Syn is a small protein composed of 140 amino acids (14 KDa) and is abundant in many regions of the brain [16,17]. α-Syn is an intrinsically disordered protein, with no defined structure. The protein has three well-characterized regions: the *N*-terminal region (1–60 amino acids), the hydrophobic region or non-A beta component of the Alzheimer’s disease amyloid (61–95 amino acids), and the *C*-terminal region (96–140 amino acids) [18,19,20,21]. The *N*-terminal region includes six copies of the repeat KTKEGV and is the fragment of the protein where familial mutations of the α-syn gene related to PD have been identified [22]. The hydrophobic region is the amyloidogenic part of the protein, related to the ability of α-syn to form fibrils in vitro and in vivo. This region is what distinguishes α-syn from the other members of the synuclein family [22,23,24,25,26]. The *C*-terminal region is rich in proline residues and has a high content of acidic amino acids, such as glutamic and aspartic acid. This contributes to the extremely low isoelectric point of α-syn (pI: 4.7) [27].

α-Syn fibril formation follows a nucleation-dependent pathway that involves many prefibrillar intermediates. The fibrillation of monomeric α-syn requires the formation of a discrete number of soluble oligomeric intermediates [28]. Oligomeric prefibrillar species include a group of intermediates of variable size and morphology [28,29,30,31,32,33,34,35,36]. Lansbury´s group has proposed that spheroidal α-syn soluble oligomers are rich in β-sheet structure and that the conversion from monomer to oligomer involves a secondary structural transition from the natively unfolded protein to predominantly β-sheets [37]. Oligomers of α-syn are formed from approximately 30 to 35 monomers and have a molecular weight of 440,000 Da. α-Syn fibrils are much larger, with about 8300 monomers per fibril and a molecular weight of around 120,000,000 Da [38]. Many reports suggest that these oligomeric species are responsible for α-syn toxicity [37,39,40,41]. It has been demonstrated that α-syn soluble oligomers disrupt membranes [37,41] and cause cell death both in vitro [30,31] and in animal models [39,40].

Therefore, processes that increase α-syn oligomer concentration, stabilize its conformation, or decrease its clearance will probably induce toxicity [35]. However, the exact role of oligomeric species in α-syn pathology is still unclear. Nevertheless, the idea that α-syn soluble oligomers are the proximal toxic species has been questioned since it has been shown that fibrils of α-syn can also induce toxicity, promote the seeding of endogenous α-syn, and may have prion-like effects [14,15,32,33,42].

## 2. Extracellular α-Synuclein

### 2.1. Putative Mechanisms of α-Syn Uptake in Cells of the Nervous System

α-Syn is a cytosolic protein that is poorly expressed in astrocytes, microglia, and oligodendrocytes [43,44]. However, it is abundantly expressed in neurons in the central nervous system (CNS). Although α-syn has no known signaling sequence, α-syn can be released from neuronal cells in small amounts via unconventional exocytosis under normal physiological conditions [45]. In pathological conditions, α-syn monomers and aggregates may be released in larger quantities and endocytosed by neighboring cells, leading to the formation of LB-like inclusions [46]. Both forms of the protein, monomeric and higher-order aggregated species, have been found in the lumen of vesicles [45].

Recent evidence showed that α-syn can propagate through neurons in the central nervous system. First, LBs were found in grafted neurons in PD patients treated with embryonic cell transplants [47,48]. Second, animal studies showed that brain inoculation of fibrillar α-syn led to the propagation of α-syn to anatomically interconnected areas of the brain, and in humans, there is evidence of trans-synaptically spreading of α-syn pathology [34,49,50,51,52]. Third, significant amounts of α-syn soluble oligomers have been detected in the plasma and cerebrospinal fluid (CSF) of patients with PD [53]. However, some observations are not consistent with this model of α-syn propagation [49,50,51,52]. Regarding Braak’s hypothesis for α-syn progression, it was found that 47% of cases with clinical symptoms of PD do not follow an ascending progression of LBs [49,50,51]. In addition, not all transplanted PD patients developed LB inclusions in the grafted neurons [54,55].

Other investigators suggested an alternative hypothesis, known as the “dual-hit”, proposing that α-syn aggregation is initiated in an extra-CNS site, such as olfactory epithelium and/or gut mucosa, in response to environmental factors, such as a neurotropic viral pathogen or a toxin [52].

What seems to be clear is that there is some level of cell loss in PD in restricted regions of the CNS and peripheral nervous system. Some hypotheses on neuronal selective vulnerability in PD have been gaining attention lately. These are mainly related to dopamine toxicity, cell iron content, and axonal arborization size [51].

Soluble monomeric α-syn can be internalized by different cells of the nervous system by multiple non-specific mechanisms. Some investigators suggested that there is a direct translocation of monomeric α-syn across the plasma membrane, avoiding the recruitment of any receptor for endocytosis [56]. A study by Outeiro’s group showed that in human neuroglioma H4 cells, the internalization of α-syn is mediated by its interaction with the plasma membrane [57]. Ahn et al. demonstrated that the 11-amino acid repeats present in the primary sequence of α-syn play a critical role in the membrane translocation of the protein [58]. However, the uptake of α-syn by clathrin-mediated endocytosis has been demonstrated in vitro in neurons, oligodendrocytes, and microglia [59,60,61,62]. However, α-syn uptake was not fully inhibited by blocking this pathway [58], suggesting alternative routes of entry, such as caveolar endocytosis. The internalization of α-syn seems to be quite different for each species (monomeric, oligomeric, or fibrillar α-syn), and there is some controversy in the literature about the exact mechanism that is behind cell internalization of α-syn. Oligomeric and fibrillar forms of the protein are internalized by cells via endocytosis, triggered by the interaction of α-syn conformers with different membrane receptors [38,61,62,63,64]. Different receptors are involved in the uptake of extracellular α-syn soluble oligomers and fibrils, and their relevance depends on the cell type. However, many questions arise: How does α-syn get inside these cells? Is there a specific receptor for α-syn? Do the different conformers of α-syn (monomer, oligomer, and fibrils) share the same receptors? The mechanisms of cellular internalization for extracellular α-syn are still not completely clarified.

### 2.2. Glial Cell Uptake of Extracellular α-Syn and Activation

#### 2.2.1. Role of Astrocytes

Astrocytes outnumber neurons in the CNS and are responsible for a wide variety of important functions, including regulation of blood flow, maintenance of the blood–brain barrier (BBB), and maintenance of the composition of the extracellular environment of ions [65]. Recent studies suggest that astrocytes play important roles in modulating neurotransmission, cell signaling, inflammation, synapse modulation, and metabolite and electrolyte homeostasis. Additional information on astrocyte–neuron metabolic functions can be found in references [66,67].

Damage to the CNS due to injury or disease may result in molecular, cellular, and functional changes in astrocytes, leading to ”reactive astrogliosis”. The process of astrocyte activation can be divided into three main stages or features: (i) morphological changes and cytokine production, (ii) cell proliferation, and (iii) cell migration. Some characteristics that describe reactive astrogliosis are: astrocyte hypertrophy, development of processes and cell proliferation, increased expression of the cytoskeleton glial fibrillary acidic protein (GFAP), and alterations in gene expression [65,66,68,69].

As well as other glial cells, astrocytes do not express α-syn or express it at very low levels [70]. However, the uptake of wild-type or mutant α-syn by astrocytes induces astrocyte reactivity, exhibiting neurotoxicity or inducing inflammation [71,72]. In the development of synucleinopathies, astrocytes may be activated, either by α-syn or by activated microglia [67,73]. Different α-syn aggregated forms activate glial cells to induce an inflammatory response [70,71]. Astrocytes exposed to neuron-derived α-syn aggregates underwent changes in their gene expression profiles with the induction of different proinflammatory cytokines and chemokines [74]. Reactive astrocytes can promote the release of proinflammatory cytokines and induce the production of reactive oxygen species, which will in turn affect neuronal survival and neuronal functions [71,74]. Oxidative stress has been implicated in the pathogenic mechanisms of PD and many other neurodegenerative diseases [75,76]. In response to oxidative stress, the levels of numerous cytoprotective products are increased via alteration of the Keap1 and Nrf2 system [77]. The formation of peroxynitrite and radicals derived from its homolysis leads to the oxidation and nitration of proteins [78,79,80]. In particular, for α-syn, the exposure of the protein to nitrating agents in vitro results in cross-linking and the formation of high-molecular-mass α-syn aggregates [81]. Pathological α-syn accumulation impairs the redox homeostasis in the nervous system; an increase in nuclear localization of NRF2 in post-mortem PD midbrain was detected [75].

The relevance of astrocytes in this scenario is also their participation in the clearance of neuronal α-syn, revealing an important role of astrocytes in the regulation of neuronal α-syn [82]. In a recently published study, it was suggested that astrocytes internalize α-syn aggregates and effectively degrade them via proteasomal and autophagic pathways [83].

**Figure 1:**
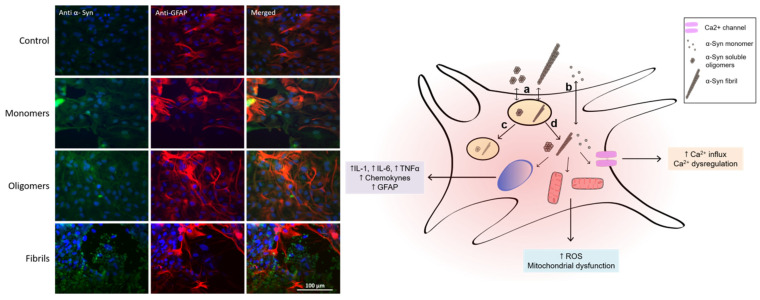
Left. Immunofluorescence for the detection of GFAP (red) and α-syn (green). Nuclei were stained with DAPI (blue). Primary culture of cortical astrocytes obtained from neonate rats was exposed for 24 h to different α-syn species. Right. Schematic representation of astrocyte and its interactions with different α-syn conformers, showing (a) endocytosis pathway of high-molecular-weight species, (b) cell membrane translocation of α-syn monomer, (c) lysosomal degradation process, and (d) cytoplasmic liberation and interaction with different cell organelles and proteins.

A relationship between mitochondrial dysfunction and α-syn has been previously reported in PD. However, most mitochondrial studies in PD were performed in neuronal cells. PD patients present an accumulation of α-syn in mitochondria and decreased complex I activity, while mice overexpressing mutated A53T α-syn have reduced complex IV activity [84,85]. Previous work indicates that astrocyte-mediated toxicity is associated with mitochondrial dysfunction in astrocytes [86]. Using the transgenic mouse model of ALS, SOD1^G93A^ mice, and mitochondrial-targeted therapies, increased survival and delayed grip strength decline were observed [87]. We showed that exposure of cultured astrocytes to different forms of α-syn (monomer, oligomer, and fibril) leads to an activated phenotype, characterized by mitochondrial dysfunction, proinflammatory cytokine production, and ROS formation [71]. Figure 1 shows the increase in GFAP immunoreactivity after the incubation of primary cortical rat astrocytes with monomeric, oligomeric, or fibrillar α-syn. It is interesting to notice that the effects in astrocytes depend on the aggregated form of α-syn [71].

Astrocytes’ cytoarchitecture dramatically changes upon exposure to oligomeric and fibrillar α-syn, with the generation of flat and polyhedral cells, retraction of the soma and nuclei, and formation of long thin processes. There is an increase in the immunostaining of the GFAP protein in astrocytes upon oligomer and fibrillar α-syn exposure along with the morphological changes. α-Syn soluble oligomers and fibrils induce the mRNA of TNF-alpha and IL-1β at similar levels to the ones obtained with LPS on astrocytes. All α-syn conformers induced the formation of reactive oxygen and nitrogen species, but only the soluble oligomeric forms led to mitochondrial dysfunction in cortical astrocytes. These activated astrocytes affect neuronal survival in co-cultures [71]. Using co-cultures of hippocampal neurons seeded on top of confluent astrocyte monolayers, we analyzed the cellular toxicity of α-syn. Pretreatment of co-cultures with different forms of α-syn significantly reduced neuron survival with respect to the control, resulting in 58%, 55%, and 15% survival for monomeric, oligomeric, and fibrillar α-syn, respectively. These results indicate that astrocytes activated by α-syn conformers can induce neuronal death or are less efficient at providing trophic support [71].

#### 2.2.2. Role of Oligodendrocytes

Oligodendrocytes are glial cells that are responsible for the myelination of axons in the CNS, having an important role in their development, maintenance, and regeneration. Oligodendrocytes undergo a complex process of proliferation, migration, and differentiation that leads to their mature form. They also provide trophic support to neurons by releasing lactate [88,89,90,91,92]. The connection between α-syn and oligodendrocytes comes from pathology [93,94]. MSA is a progressive and severe neurodegenerative disorder that is clinically characterized by variable degrees of parkinsonism, cerebellar ataxia, and dysautonomia [95]. The hallmark of the disease is the presence of glial cytoplasmic inclusions (GCIs), which are intracellular protein aggregates, mainly composed of α-syn, located in oligodendrocytes [7,96]. Further components of GCIs are ubiquitin and other proteins, such as leucine-rich repeat serine/threonine-protein LRRK2, heat shock proteins, microtubule-associated protein tau, and prion disease-linked 14-3-3 protein, among others [96]. Analysis of single-nucleotide polymorphisms (SNPs) in the *SNCA* gene, the gene that encodes for α-syn, has identified an association between certain α-syn SNPs and an increased risk for the development of MSA [97].

Even though α-syn mRNAs and protein were detected in rat brain oligodendrocytes [98], α-syn expression was not detected in oligodendrocytes from healthy and MSA human brains [43]. This implies that endogenous α-syn is not enough for the formation of intracellular aggregates associated with pathology. It is worth mentioning that no accompanying neuronal Lewy pathology is observed in MSA patients, and in PD patients, α-syn forms aggregates mainly in neurons but to a lesser extent in oligodendrocytes [99]. Oligodendrocytes are able to uptake neuronal α-syn and then neuronal secreted α-syn, which could contribute to or initiate the cytoplasmic inclusions found in oligodendrocytes in MSA. Kisos et al. showed that oligodendrocytes are capable of taking up α-syn from their environment, either from a medium conditioned by neuronal cells or from their growth medium supplemented with recombinant human α-syn. They demonstrated that the uptake by oligodendrocytes is dependent on clathrin expression [98]. In addition, Ihse et al. showed that α-syn fibrils secreted from neurons are internalized by oligodendrocytes in a process mediated by cell surface heparan sulfate proteoglycans [64]. Although PD and MSA can be clinically alike (regarding motor symptoms), one of the main pathological differences between them is the distribution of neuronal loss (in discrete regions in PD and widespread in MSA). This may imply different progression mechanisms for both diseases, where oligodendroglial cells could have a decisive role or other α-syn family members may be involved, as suggested by McCann et al. [93]. It is also worth mentioning that there is no evidence of prion-like propagation of α-syn in oligodendrocytes [100]. An important correlation was found between cytoplasmic LB inclusions or LB neurites and oligodendroglial α-synucleinopathy, pointing towards a strong PD and MSA relation [94].

In response to cellular stress, oligodendrocytes suffer from oligodendroglial dysfunction. This is a phenomenon characterized by increased cell vulnerability and leads to cellular dysfunction, demyelination, and eventually, cell death. The accumulation of intracellular α-syn in oligodendrocytes leads to oligodendroglial dysfunction, also associated with neuroinflammation and demyelination. Cell death and neuronal loss occur via diverse mechanisms, such as the induction of oxidative stress, cytokine production, and altered cell–extracellular matrix interactions (impaired cell adhesion properties). It was demonstrated that in a transgenic mouse model expressing α-syn in oligodendrocytes (under the control of the MBP promoter), there was a decrease in the expression of neurotrophic factors, especially glial-derived neurotrophic factor (GDNF) released from oligodendrocytes, providing new insight into the possible pathogenic mechanisms of oligodendroglial α-synucleinopathies [101].

Oligodendrocytes’ ability to propagate misfolded α-syn species can be explored using different animal models by injecting external α-syn aggregates into peripheral tissues and by analyzing the appearance of protein inclusions in white matter in the brain and spinal cord [102,103]. Using these models, it was demonstrated that muscular injection of α-syn PFF into transgenic M83^+/+^ hind limbs produces the spatio-temporal progression of neuronal α-syn inclusions and the spatio-temporal progression of oligodendrocyte α-syn inclusions, with the latter being more widespread than the former [103]. The same animal model was used to investigate nerve conduction and axon degeneration, and it was suggested that the propagation of α-syn pathology could be preferential in myelinated axons [42]. Although nerve axons are myelinated by Schwann cells (and not by oligodendrocytes), oligodendrocytes’ proximity during axon degeneration and retrograde axonal transport could be sufficient to cause the cell-to-cell spreading of misfolded α-syn.

#### 2.2.3. Role of Microglia

Microglia are phagocytic cells of the brain that regulate brain development, the maintenance of neuronal networks, and injury repair. These cells release trophic factors, including brain-derived neurotrophic factor (BDNF) and GDNF [104]. During development, microglia help shape neural circuits and, in response to CNS injury, are responsible for the phagocytosis and elimination of microbes, dead cells, and protein aggregates [105].

Accumulation and activation of microglia in the CNS have been termed microgliosis. Activated microglia change the movement of their processes from undirected to targeted towards the injured site [106]. Microglial cells express a wide range of immune receptors, such as pattern recognition receptors (PRRs) that recognize pathogen-associated molecular patterns (PAMPs) or tissue damage–associated molecular patterns (DAMPs). Microglia PRRs include toll-like receptors (TLRs), particularly TLR4 and TLR1/2 and their co-receptors [107]. They also express receptors for phagocytosis and endocytosis pathways, chemokine receptors, and the lymphocyte-activation gene 3 (LAG3) receptor, which is discussed in the next section [104,105,108].

The relationship between α-syn, microglia, and disease arises from the observation that PD patients demonstrate a marked increase in activated microglia with increased expression and concentration of proinflammatory cytokines [104,108,109]. In addition, reactive microglia assemble close to LBs in PD patients [110]. Moreover, α-syn leads to microglial activation in mouse models of protein overexpression prior to dopaminergic neuronal death [111].

Microglia exposed to α-syn soluble oligomers upregulate the expression of genes encoding TLR and the proinflammatory cytokines TNF-α and IL-1β [112]. They also present morphological changes indicative of microglial activation. Microglial activation is also associated with the generation of reactive oxygen and nitrogen species [113]. In the substantia nigra pars compacta of MSA mice, increased expression of inducible nitric oxide synthase was detected [114]. The activation of microglia and the proinflammatory response produced can accelerate the loss of dopaminergic neurons and the progression of synucleinopathies [105]. The different conformers of α-syn, mainly soluble oligomers, induce a specific response. In contrast, monomeric α-syn does not induce detectable microglial activation but promotes microglial phagocytosis [115]. α-Syn preformed fibrils (PFFs) also induced the activation of microglia [116]. Information from proteomics indicates that α-syn PFF leads to expression changes of microglial genes involved in RNA binding, mitochondrial stress, and lysosomal and autophagic functions, shedding light on the pathways involved in α-syn PFF activation of microglia [117].

Kim et al. demonstrated that microglial uptake of neuronal α-syn depends on TLR2. However, it is proposed that the uptake of α-syn can be mediated not only by TLR2 but also by other cell surface receptors [118]. The activation of microglia induced by α-syn, particularly higher-ordered oligomeric species (>720 KDa), also relies on TLR2 [60]. The mechanism of α-syn oligomeric activation of microglia depends on a MyD88-dependent TLR1/2 pathway [60]. This pathway is also highly sensitive to the conformation of the protein; purely monomeric or fibrillar α-syn cannot activate TLR2. In addition, microglial TLR4 is involved in the uptake and clearance of extracellular α-syn [61].

## 3. Receptors for Extracellular α-Syn

**Figure 2:**
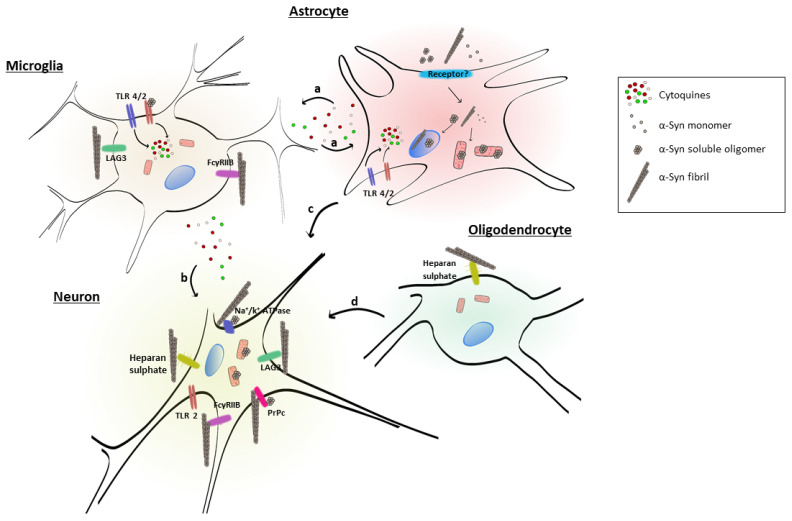
Illustration of glial cells and neurons and their interactions with different α-syn conformers, showing (a) liberation of proinflammatory cytokines from astroglial and microglial cells, contributing to their activation, (b) liberation of proinflammatory cytokines from microglial cells that affect neurons, (c) activation of astrocytes, impairing trophic support to neurons, and (d) myelination deficiency, negatively affecting axonal conduction and neuronal function.

Table 1 and Figure 2 summarize the extracellular receptors discussed here, identifying the α-syn conformer and the cell type involved. The LAG3 receptor belongs to the immunoglobulin superfamily. It is highly expressed in some immune organs, including the spleen and the thymus, and also in the central nervous system [119,120]. LAG3 can be expressed on neuronal cells and in microglia [121]. This receptor regulates T cell immune responses and immune homeostasis, mainly by inhibiting T cell activation and proliferation. LAG3 demonstrated the highest ratio of selectivity for α-syn PFF over monomeric α-syn. The internalization of α-syn PFF in neurons involves LAG3, since the deletion of LAG3 reduces the endocytosis of α-syn PFF, and this is specific for α-syn PFF. Neuron-to-neuron transmission of α-syn and the induction of neurotoxicity are attenuated by the deletion of LAG3 [63]. The lack of LAG3 delayed the α-syn PFF-induced loss of dopamine neurons, as well as biochemical and behavioral deficits in vivo [63]. Some authors described an impairment in the pole test of animals injected with PFF, which was also prevented by LAG3 deletion [63].

These data suggest that extracellular α-syn fibrils can bind to LAG3 and contribute to protein-induced dopaminergic neuronal loss and neurotoxicity [63]. This receptor may play a role in α-syn spreading pathology and neurodegeneration in PD and could be considered as a therapeutic target to avoid α-syn pathology.

The cellular prion protein (PrPc) is another protein that contributes to α-syn cell internalization. PrPc is a surface protein anchored to the cell membrane through a C-terminal glycosylphosphatidylinositol (GPI) moiety. Among its variety of known functions, it is involved in the cell cycle and proliferation and also in copper homeostasis and neuroprotection. It was recently suggested to have a role as a receptor for amyloid-β (Aβ) oligomers and to mediate Aβ-induced synaptic dysfunction [126]. Thus, many investigators have studied whether the function of PrPc as a receptor for aggregated proteins [127] could also be attributable to α-syn species.

Using a murine neuroblastoma cell line (N2a) and a model of mouse primary cortical neurons constitutively expressing PrPc, it was determined that the presence of PrPC is required for the uptake of fibrillar forms of α-syn [122]. Using mice hippocampal slices exposed to extracellular α-syn soluble oligomers, it was shown that oligomers also interacted physically with PrPc [123]. The interaction between α-syn and PrPc causes synaptic dysfunction via a signaling cascade acting through the phosphorylation of Fyn kinase and the activation of the N-methyl-D-aspartate receptor [123]. This could be the signaling cascade that leads to synaptic dysfunction in the hippocampus [123].

Proteoglycans are known for interacting with different ligands and facilitating the cellular internalization of amyloid proteins, particularly Aβ, tau, and the prion protein [128]. They are glycoproteins that contain one or more sulfated glycosaminoglycan (GAG) chains [129]. The interaction occurs between negatively charged groups in GAG chains with positively charged amino acids in the amyloid protein [127]. Ihse et al. demonstrated that the internalization of α-syn fibrils in neurons depends on their interaction with heparan sulfate [64]. The investigators proposed that the process of fibril formation might expose positively charged domains that can interact with heparan sulfate. Soluble α-syn oligomers do not enter the cell using this pathway [64]. This heparan sulfate internalization pathway for α-syn fibrils is used by non-immune brain cells such as neurons and oligodendrocytes, while it is less important for astrocytes and microglia [64].

TLRs belong to the family of pattern recognition receptors and are crucial players in the innate immune response. They are expressed in astrocytes and innate immune system cells, including microglial cells [130,131]. The link between TLR and α-syn is based on the upregulation of TLRs observed in transgenic mouse models of MSA-like neuropathology and human MSA brains [113,132,133].

Initially, it was suggested that TLR4 mediates the incorporation of α-syn into astrocytes, but this is not the case. After astrocyte incubation with α-syn, α-syn was detected in the cytoplasm, independent of TLR4 expression [118,134]. The upregulation of TLR4 in microglia allows the clearance of extracellular α-syn [61]. In astrocytes, α-syn uptake is independent of TLR4, but extracellular α-syn can activate proinflammatory TLR4 pathways in these cells [135]. However, in microglia, α-syn incorporation into the cell depends on TLR4; thus, different mechanisms control the uptake of α-syn in microglia and astroglia [134]. Stimulation of TLR2 in neurons, astrocytes, and microglia increased the uptake of α-syn fibrils. However, α-syn oligomers are better inductors of neuroinflammation in comparison with monomers or fibrils, acting as TLR2 agonists [60].

Thus, neurons, astrocytes, and microglia all presented increased α-syn fibril uptake following innate immune receptor stimulation, although the underlying mechanisms of α-syn degradation in these cells appear to be different.

The a3-subunit of the neuronal pump Na^+^/K^+^-ATPase has been recently identified as another receptor for α-syn conformers [136]. In recent work using pull-down immunoprecipitation, it was found that the neuron-specific a3-subunit of the plasma membrane-enriched enzyme Na^+^/K^+^-ATPase was the only transmembrane protein identified to interact with both forms of the protein, oligomeric and fibrillar α-syn [124]. The strength of the interaction depends on the species of α-syn, with fibrils being the strongest, soluble oligomers being weaker, and monomers presenting no interaction [124]. It was hypothesized that the interaction of this receptor with α-syn induces an alteration in the pumping activity of Na^+^/K^+^-ATPase, impairing the maintenance of the Na^+^ gradient and thus disrupting neuronal function [124].

The FcƔRIIB receptor binds immunoglobulin G (IgG) with low affinity and interacts with immune complexes only at a physiological concentration of the antibody. FcƔRIIB is expressed in neurons and transmits signals from extracellular α-syn fibrils to the cytoplasm. Choi et al. demonstrated that FcƔRIIB expressed in microglia binds to α-syn fibrils [125], and this inhibits microglial phagocytosis. However, no differences in the ability of BV2 microglial cells to phagocytose α-syn in relation to the aggregation state of the protein were reported [131]. There is some evidence that α-syn may interfere with phagocytosis in microglia, but the mechanism behind these phenomena is still unclear [137].

## 4. Conclusions

α-Syn conformers are taken up by different mechanisms; the monomeric protein is translocated across the plasma membrane, while oligomeric and fibrillar α-syn require an extracellular receptor for their uptake. Glial cells’ role in this process could be attributable to the clearing of α-syn, and this might be a mechanism for preventing neurons from exposure to potentially toxic α-syn.

The interaction of α-syn with LAG3, PrPC, and heparan sulfate mediates the uptake of α-syn fibrils but not of the monomeric form of the protein. These receptors are relevant mainly in neuronal cells. In microglia and also in some neuron types, α-syn internalization depends on TLRs, particularly TLR2 and TLR4.

Under local stress conditions, α-syn has been detected in the extracellular space due to neuronal death or exocytosis. The secretion of α-syn would be a beneficial process for neurons, but it may enhance the progression of PD and related synucleinopathies. Extracellular α-syn can be taken up by surrounding neurons or by glial cells. Most of the studies on α-syn have focused on neuronal cells, but glia have an important role in synucleinopathies and require further investigation. It is still uncertain when extracellular α-syn is no longer being cleared from the neuropil, mainly by microglial cells, and starts to induce microgliosis, astrogliosis, or oligodendrocyte activation. Is there a concentration threshold or a conformational-dependent process? Or are both phenomena needed? Additional factors are probably needed to allow the accumulation of oligomeric and fibrillar α-syn. The epidemiology of PD points to additional external and genetic effectors, such as environmental toxins or mutated or overexpressed wild-type α-syn. It is highly possible that the involvement of some of these factors, acting together, contributes to the progression of synucleinopathies.

## Figures and Tables

**Table 1:** Glial and neuronal receptors involved in extracellular α-syn uptake in the CNS.

Receptor	α-Syn Conformer	Cell Type	Reference
Lymphocyte-activation gene 3 (LAG3)	Fibrils	Neuron, microglia	[63,121]
Cellular prion protein (PrPc)	Fibrils, soluble oligomers	Neurons	[122,123]
Heparan sulfate	Fibrils	Neuron, oligodendrocytes	[64]
Toll-like receptor 4 (TLR4)	n.d.	Microglia	[61]
Toll-like receptor 2 (TLR2)	Soluble oligomers	Microglia	[118]
a3-subunit of Na^+^/K^+^-ATPase	Fibrils, soluble oligomers	Neurons	[124]
FcƔRIIB	Fibrils	Microglia, neurons	[125]

## References

[1] Parkinson J. (2002). “An essay on the shaking palsy” 200 years old. J. Neuropsychiatry Clin. Neurosci..

[2] Poewe W., Seppi K., Tanner C.M., Halliday G.M., Brundin P., Volkmann J., Schrag A.E., Lang A.E. (2017). Parkinson disease. Nat. Rev. Dis. Prim..

[3] Lees A.J., Hardy J., Revesz T. (2009). Parkinson’s disease. Lancet.

[4] Dauer W., Przedborski S. (2003). Parkinson’s Disease: Mechanisms and Models. Camb. Companion Philos. Biol..

[5] Kalia L.V., Lang A.E. (2015). Parkinson’s disease. Lancet.

[6] Baba M., Nakajo S., Tu P.H., Tomita T., Nakaya K., Lee V.M., Trojanowski J.Q., Iwatsubo T. (1998). Aggregation of alpha-synuclein in Lewy bodies of sporadic Parkinson’s disease and dementia with Lewy bodies. Am. J. Pathol..

[7] Spillantini M.G., Crowther R.A., Jakes R., Cairns N.J., Lantos P.L., Goedert M. (1998). Filamentous alpha-synuclein inclusions link multiple system atrophy with Parkinson’s disease and dementia with Lewy bodies. Neurosci. Lett..

[8] Zarranz J.J., Alegre J., Gomez-Esteban J.C., Lezcano E., Ros R., Ampuero I., Vidal L., Hoenicka J., Rodriguez O., Atares B. (2004). The new mutation, E46K, of alpha-synuclein causes Parkinson and Lewy body dementia. Ann. Neurol..

[9] Singleton A.B., Farrer M., Johnson J., Singleton A., Hague S., Kachergus J., Hulihan M., Peuralinna T., Dutra A., Nussbaum R. (2003). alpha-Synuclein locus triplication causes Parkinson’s disease. Science.

[10] Blesa J., Przedborski S. (2014). Parkinson’s disease: Animal models and dopaminergic cell vulnerability. Front. Neuroanat..

[11] Dehay B., Fernagut P.O. (2016). Alpha-synuclein-based models of Parkinson’s disease. Rev. Neurol..

[12] Duty S., Jenner P. (2011). Animal models of Parkinson’s disease: A source of novel treatments and clues to the cause of the disease. Br. J. Pharmacol..

[13] Koprich J.B., Kalia L.V., Brotchie J.M. (2017). Animal models of α-synucleinopathy for Parkinson disease drug development. Nat. Rev. Neurosci..

[14] Uchihara T., Giasson B.I. (2016). Propagation of alpha-synuclein pathology: Hypotheses, discoveries, and yet unresolved questions from experimental and human brain studies. Acta Neuropathol..

[15] Schaser A.J., Stackhouse T.L., Weston L.J., Kerstein P.C., Osterberg V.R., López C.S., Dickson D.W., Luk K.C., Meshul C.K., Woltjer R.L. (2020). Trans-synaptic and retrograde axonal spread of Lewy pathology following pre-formed fibril injection in an in vivo A53T alpha-synuclein mouse model of synucleinopathy. Acta Neuropathol. Commun..

[16] Lee V.M., Trojanowski J.Q. (2006). Mechanisms of Parkinson’s disease linked to pathological alpha-synuclein: New targets for drug discovery. Neuron.

[17] Savitt J.M., Dawson V.L., Dawson T.M. (2006). Diagnosis and treatment of Parkinson disease: Molecules to medicine. J. Clin. Investig..

[18] Chandra S., Chen X., Rizo J., Jahn R., Sudhof T.C. (2003). A broken alpha -helix in folded alpha -Synuclein. J. Biol. Chem..

[19] Davidson W.S., Jonas A., Clayton D.F., George J.M. (1998). Stabilization of alpha-synuclein secondary structure upon binding to synthetic membranes. J. Biol. Chem..

[20] George J.M. (2002). The synucleins. Genome Biol..

[21] Kahle P.J., Haass C., Kretzschmar H.A., Neumann M. (2002). Structure/function of alpha-synuclein in health and disease: Rational development of animal models for Parkinson’s and related diseases. J. Neurochem..

[22] Lavedan C. (1998). The synuclein family. Genome Res..

[23] Culvenor J.G., McLean C.A., Cutt S., Campbell B.C., Maher F., Jakala P., Hartmann T., Beyreuther K., Masters C.L., Li Q.X. (1999). Non-Abeta component of Alzheimer’s disease amyloid (NAC) revisited. NAC and alpha-synuclein are not associated with Abeta amyloid. Am. J. Pathol..

[24] el-Agnaf O.M., Irvine G.B. (2002). Aggregation and neurotoxicity of alpha-synuclein and related peptides. Biochem. Soc. Trans..

[25] Ferrer I. (2001). Alpha-synucleinopathies. Neurologia.

[26] Ueda K., Fukushima H., Masliah E., Xia Y., Iwai A., Yoshimoto M., Otero D.A., Kondo J., Ihara Y., Saitoh T. (1993). Molecular cloning of cDNA encoding an unrecognized component of amyloid in Alzheimer disease. Proc. Natl. Acad. Sci. USA.

[27] Hoyer W., Antony T., Cherny D., Heim G., Jovin T.M., Subramaniam V. (2002). Dependence of alpha-synuclein aggregate morphology on solution conditions. J. Mol. Biol..

[28] Goedert M., Masuda-Suzukake M., Falcon B. (2017). Like prions: The propagation of aggregated tau and α-synuclein in neurodegeneration. Brain.

[29] Conway K.A., Harper J.D., Lansbury P.T. (2000). Fibrils formed in vitro from alpha-synuclein and two mutant forms linked to Parkinson’s disease are typical amyloid. Biochemistry.

[30] Kayed R., Sokolov Y., Edmonds B., McIntire T.M., Milton S.C., Hall J.E., Glabe C.G. (2004). Permeabilization of lipid bilayers is a common conformation-dependent activity of soluble amyloid oligomers in protein misfolding diseases. J. Biol. Chem..

[31] Kim H.Y., Cho M.K., Kumar A., Maier E., Siebenhaar C., Becker S., Fernandez C.O., Lashuel H.A., Benz R., Lange A. (2009). Structural properties of pore-forming oligomers of alpha-synuclein. J. Am. Chem. Soc..

[32] Danzer K.M., Haasen D., Karow A.R., Moussaud S., Habeck M., Giese A., Kretzschmar H., Hengerer B., Kostka M. (2007). Different species of alpha-synuclein oligomers induce calcium influx and seeding. J. Neurosci..

[33] Kayed R., Head E., Thompson J.L., McIntire T.M., Milton S.C., Cotman C.W., Glabe C.G. (2003). Common structure of soluble amyloid oligomers implies common mechanism of pathogenesis. Science.

[34] Volpicelli-Daley L.A., Luk K.C., Lee V.M. (2014). Addition of exogenous alpha-synuclein preformed fibrils to primary neuronal cultures to seed recruitment of endogenous alpha-synuclein to Lewy body and Lewy neurite-like aggregates. Nat. Protoc..

[35] Lashuel H.A., Overk C.R., Oueslati A., Masliah E. (2013). The many faces of α-synuclein: From structure and toxicity to therapeutic target. Nat. Rev. Neurosci..

[36] Alam P., Bousset L., Melki R., Otzen D.E. (2019). A-Synuclein Oligomers and Fibrils: A Spectrum of Species, a Spectrum of Toxicities. J. Neurochem..

[37] Volles M.J., Lee S.J., Rochet J.C., Shtilerman M.D., Ding T.T., Kessler J.C., Lansbury P.T. (2001). Vesicle permeabilization by protofibrillar alpha-synuclein: Implications for the pathogenesis and treatment of Parkinson’s disease. Biochemistry.

[38] Pieri L., Madiona K., Melki R. (2016). Structural and functional properties of prefibrillar alpha-synuclein oligomers. Sci. Rep..

[39] Karpinar D.P., Balija M.B., Kugler S., Opazo F., Rezaei-Ghaleh N., Wender N., Kim H.Y., Taschenberger G., Falkenburger B.H., Heise H. (2009). Pre-fibrillar alpha-synuclein variants with impaired beta-structure increase neurotoxicity in Parkinson’s disease models. EMBO J..

[40] Winner B., Jappelli R., Maji S.K., Desplats P.A., Boyer L., Aigner S., Hetzer C., Loher T., Vilar M., Campioni S. (2011). In vivo demonstration that alpha-synuclein oligomers are toxic. Proc. Natl. Acad. Sci. USA.

[41] Luth E.S., Stavrovskaya I.G., Bartels T., Kristal B.S., Selkoe D.J. (2014). Soluble, prefibrillar α-synuclein oligomers promote complex I-dependent, Ca2+-induced mitochondrial dysfunction. J. Biol. Chem..

[42] Ferreira N., Gonçalves N.P., Jan A., Jensen N.M., Van Der Laan A., Mohseni S., Vægter C.B., Jensen P.H. (2021). Trans—Synaptic spreading of alpha—Synuclein pathology through sensory afferents leads to sensory nerve degeneration and neuropathic pain. Acta Neuropathol. Commun..

[43] Miller D.W., Johnson J.M., Solano S.M. (2005). Absence of a -synuclein mRNA expression in normal and multiple system atrophy oligodendroglia. J. Neural Transm..

[44] Booth H.D.E., Hirst W.D., Wade-Martins R. (2017). The Role of Astrocyte Dysfunction in Parkinson’s Disease Pathogenesis. Trends Neurosci..

[45] Lee H.-J., Patel S., Lee S.-J. (2005). Intravesicular localization and exocytosis of alpha-synuclein and its aggregates. J. Neurosci..

[46] Jang A., Lee H.-J., Suk J.-E., Jung J.-W., Kim K.-P., Lee S.-J. (2010). Non-classical exocytosis of alpha-synuclein is sensitive to folding states and promoted under stress conditions. J. Neurochem..

[47] Li J.Y., Englund E., Holton J.L., Soulet D., Hagell P., Lees A.J., Lashley T., Quinn N.P., Rehncrona S., Bjorklund A. (2008). Lewy bodies in grafted neurons in subjects with Parkinson’s disease suggest host-to-graft disease propagation. Nat. Med..

[48] Kordower J.H., Chu Y., Hauser R.A., Freeman T.B., Olanow C.W. (2008). Lewy body-like pathology in long-term embryonic nigral transplants in Parkinson’s disease. Nat. Med..

[49] Surmeier D.J., Obeso J.A., Halliday G.M. (2017). Selective neuronal vulnerability in Parkinson disease. Nat. Rev. Neurosci..

[50] Giguère N., Nanni S.B., Trudeau L.E. (2018). On cell loss and selective vulnerability of neuronal populations in Parkinson’s disease. Front. Neurol..

[51] Engelender S., Isacson O. (2017). The Threshold Theory for Parkinson’s Disease. Trends Neurosci..

[52] Jan A., Gonçalves N.P., Vaegter C.B., Jensen P.H., Ferreira N. (2021). The prion-like spreading of alpha-synuclein in parkinson’s disease: Update on models and hypotheses. Int. J. Mol. Sci..

[53] El-Agnaf O.M.A., Salem S.A., Paleologou K.E., Curran M.D., Gibson M.J., Court J.A., Schlossmacher M.G., Allsop D. (2006). Detection of oligomeric forms of α-synuclein protein in human plasma as a potential biomarker for Parkinson’s disease. FASEB J..

[54] Mendez I., Vĩuela A., Astradsson A., Mukhida K., Hallett P., Robertson H., Tierney T., Holness R., Dagher A., Trojanowski J.Q. (2008). Dopamine neurons implanted into people with Parkinson’s disease survive without pathology for 14 years. Nat. Med..

[55] Hallett P.J., Cooper O., Sadi D., Robertson H., Mendez I., Isacson O. (2014). Long-Term Health of Dopaminergic Neuron Transplants in Parkinson’s Disease Patients. Cell Rep..

[56] Lee S.J. (2008). Origins and effects of extracellular alpha-synuclein: Implications in Parkinson’s disease. J. Mol. Neurosci..

[57] Masaracchia C., Hnida M., Gerhardt E., Lopes da Fonseca T., Villar-Pique A., Branco T., Stahlberg M.A., Dean C., Fernández C.O., Milosevic I. (2018). Membrane binding, internalization, and sorting of alpha-synuclein in the cell. Acta Neuropathol. Commun..

[58] Ahn K.J., Paik S.R., Chul K., Kim J. (2006). Amino acid sequence motifs and mechanistic features of the membrane translocation of a -synuclein. J. Neurochem..

[59] Liu J., Zhou Y., Wang Y., Fong H., Murray T.M., Zhang J. (2007). Identification of proteins involved in microglial endocytosis of α-synuclein. J. Proteome Res..

[60] Daniele S.G., Béraud D., Davenport C., Cheng K., Yin H., Maguire-Zeiss K.A. (2015). Activation of MyD88-dependent TLR1/2 signaling by misfolded α-synuclein, a protein linked to neurodegenerative disorders. Sci. Signal..

[61] Stefanova N., Fellner L., Reindl M., Masliah E., Poewe W., Wenning G.K. (2011). Toll-like receptor 4 promotes α-synuclein clearance and survival of nigral dopaminergic neurons. Am. J. Pathol..

[62] Choi Y.R., Kang S.J., Kim J.M., Lee S.J., Jou I., Joe E.H., Park S.M. (2015). FcγRIIB mediates the inhibitory effect of aggregated α-synuclein on microglial phagocytosis. Neurobiol. Dis..

[63] Mao X., Ou M.T., Karuppagounder S.S., Kam T.-I., Yin X., Xiong Y., Ge P., Essien Umanah G., Brahmachari S., Shin J.-H. (2016). Pathological α-synuclein transmission initiated by binding lymphocyte-activation gene 3. Science.

[64] Ihse E., Yamakado H., Van Wijk X.M., Lawrence R., Esko J.D. (2017). Cellular internalization of alpha- synuclein aggregates by cell surface heparan sulfate depends on aggregate conformation and cell type. Sci. Rep..

[65] Sofroniew M.V., Vinters H.V. (2010). Astrocytes: Biology and pathology. Acta Neuropathol..

[66] Maragakis N.J., Rothstein J.D. (2006). Mechanisms of Disease: Astrocytes in neurodegenerative disease. Nat. Clin. Pract. Neurol..

[67] Mavroeidi P., Xilouri M. (2021). Neurons and glia interplay in α-synucleinopathies. Int. J. Mol. Sci..

[68] Pekny M., Pekna M., Messing A., Steinhäuser C., Lee J.M., Parpura V., Hol E.M., Sofroniew M.V., Verkhratsky A. (2016). Astrocytes: A central element in neurological diseases. Acta Neuropathol..

[69] Sofroniew M. (2009). V Molecular dissection of reactive astrogliosis and glial scar formation. Trends Neurosci..

[70] Tanji K., Imaizumi C.A.T., Yoshida H., Mori F., Yoshimoto M., Satoh K., Wakabayashi K. (2001). Expression of a-synuclein in a human glioma cell line and its up-regulation by interleukin-1 beta. Neuroreport.

[71] Chavarría C., Rodríguez-bottero S., Quijano C., Cassina P., Souza J.M. (2018). Impact of monomeric, oligomeric and fibrillar alpha-synuclein on astrocyte reactivity and toxicity to neurons. Biochem. J..

[72] Roodveldt C., Christodoulou J., Dobson C.M. (2008). Immunological features of α-synuclein in Parkinson’s disease. J. Cell. Mol. Med..

[73] Brück D., Wenning G.K., Stefanova N., Fellner L. (2016). Glia and alpha-synuclein in neurodegeneration: A complex interaction. Neurobiol. Dis..

[74] Lee H.J., Kim C., Lee S.J. (2010). Alpha-synuclein stimulation of astrocytes: Potential role for neuroinflammation and neuroprotection. Oxid. Med. Cell. Longev..

[75] Delaidelli A., Richner M., Jiang L., van der Laan A., Bergholdt Jul Christiansen I., Ferreira N., Nyengaard J.R., Vægter C.B., Jensen P.H., Mackenzie I.R. (2021). α-Synuclein pathology in Parkinson disease activates homeostatic NRF2 anti-oxidant response. Acta Neuropathol. Commun..

[76] Schipper H.M., Liberman A., Stopa E.G. (1998). Neural heme oxygenase-1 expression in idiopathic Parkinson’s disease. Exp. Neurol..

[77] Tanji K., Maruyama A., Odagiri S., Mori F., Itoh K., Kakita A., Takahashi H., Wakabayashi K. (2013). Keap1 is localized in neuronal and glial cytoplasmic inclusions in various neurodegenerative diseases. J. Neuropathol. Exp. Neurol..

[78] Ferrer-sueta G., Campolo N., Trujillo M., Bartesaghi S., Carballal S., Romero N., Alvarez B., Radi R. (2018). Biochemistry of Peroxynitrite and Protein Tyrosine Nitration. Chem. Rev..

[79] Radi R. (2004). Nitric oxide, oxidants, and protein tyrosine nitration. Proc. Natl. Acad. Sci. USA.

[80] Chavarría C., Souza J.M. (2013). Oxidation and nitration of alpha-synuclein and their implications in neurodegenerative diseases. Arch. Biochem. Biophys..

[81] Souza J.M., Giasson B.I., Chen Q., Lee V.M.Y., Ischiropoulos H. (2000). Dityrosine cross-linking promotes formation of stable α-synuclein polymers. Implication of nitrative and oxidative stress in the pathogenesis of neurodegenerative synucleinopathies. J. Biol. Chem..

[82] Tsunemi T., Ishiguro Y., Yoroisaka A., Valdez C., Miyamoto K., Ishikawa K., Saiki S., Akamatsu W., Hattori N., Krainc D. (2020). Astrocytes Protect Human Dopaminergic Neurons from a -Synuclein Accumulation and Propagation. J. Neurosci..

[83] Domenico A., Carola G., Calatayud C., Pons-espinal M., Richaud-patin Y., Fernandez-carasa I., Gut M., Faella A., Parameswaran J., Soriano J. (2019). Patient-Specific iPSC-Derived Astrocytes Contribute to Non-Cell-Autonomous Neurodegeneration in Parkinson’s Disease. Stem Cell Rep..

[84] Martin L.J., Pan Y., Price A.C., Sterling W., Copeland N.G., Jenkins N.A., Price D.L., Lee M.K. (2006). Parkinson’s disease α-synuclein transgenic mice develop neuronal mitochondrial degeneration and cell death. J. Neurosci..

[85] Devi L., Raghavendran V., Prabhu B.M., Avadhani N.G., Anandatheerthavarada H.K. (2008). Mitochondrial import and accumulation of alpha-synuclein impair complex I in human dopaminergic neuronal cultures and Parkinson disease brain. J. Biol. Chem..

[86] Cassina P., Cassina A., Pehar M., Castellanos R., Gandelman M., de Leon A., Robinson K.M., Mason R.P., Beckman J.S., Barbeito L. (2008). Mitochondrial dysfunction in SOD1G93A-bearing astrocytes promotes motor neuron degeneration: Prevention by mitochondrial-targeted antioxidants. J. Neurosci..

[87] Miquel E., Cassina A., Martinez-Palma L., Souza J.M., Bolatto C., Rodriguez-Bottero S., Logan A., Smith R.A., Murphy M.P., Barbeito L. (2014). Neuroprotective effects of the mitochondria-targeted antioxidant MitoQ in a model of inherited amyotrophic lateral sclerosis. Free Radic. Biol. Med..

[88] Konno M., Hasegawa T., Baba T., Miura E., Sugeno N., Kikuchi A., Fiesel F.C., Sasaki T., Aoki M., Itoyama Y. (2012). Suppression of dynamin GTPase decreases -synuclein uptake by neuronal and oligodendroglial cells: A potent therapeutic target for synucleinopathy. Mol. Neurodegener..

[89] Reyes J.F., Rey N.L., Bousset L., Melki R., Brundin P., Angot E. (2014). Alpha-synuclein transfers from neurons to oligodendrocytes. Glia.

[90] Lee Y., Morrison B.M., Li Y., Lengacher S., Farah M.H., Hoffman P.N., Liu Y., Tsingalia A., Jin L., Zhang P. (2012). Oligodendroglia metabolically support axons and contribute to neurodegeneration. Nature.

[91] Funfschilling U., Supplie L.M., Mahad D., Boretius S., Aiman S., Edgar J., Brinkmann B.G., Kassmann C.M., Tzvetanova I.D., Sereda W. (2013). Glycolytic oligodendrocytes maintain myelin and long-term axonal integrity. Nature.

[92] Philips T., Rothstein J.D. (2017). Oligodendroglia: Metabolic supporters of neurons. J. Clin. Investig..

[93] McCann H., Stevens C.H., Cartwright H., Halliday G.M. (2014). α-Synucleinopathy phenotypes. Park. Relat. Disord..

[94] Seidel K., Mahlke J., Siswanto S., Krüger R., Heinsen H., Auburger G., Bouzrou M., Grinberg L.T., Wicht H., Korf H.W. (2015). The brainstem pathologies of Parkinson’s disease and dementia with lewy bodies. Brain Pathol..

[95] Gilman S., Low P.A., Quinn N., Albanese A., Fowler C.J., Kaufmann H., Klockgether T., Lang A.E., Lantos P.L., Litvan I. (1999). Consensus statement on the diagnosis of multiple system atrophy. J. Neurol. Sci..

[96] Mccormack A., Chegeni N., Chegini F., Colella A., Power J., Keating D., Chataway T. (2016). Purification of α-synuclein containing inclusions from human post mortem brain tissue. J. Neurosci. Methods.

[97] Scholz S.W., Houlden H., Schulte C., Sharma M., Li A., Berg D., Melchers A., Paudel R., Gibbs J.R., Simon-Sanchez J. (2009). SNCA variants are associated with increased risk for multiple system atrophy. Ann. Neurol..

[98] Kisos H., Pukaß K., Ben-hur T., Richter-landsberg C., Sharon R. (2012). Increased Neuronal a -Synuclein Pathology Associates with Its Accumulation in Oligodendrocytes in Mice Modeling a -Synucleinopathies. PLoS ONE.

[99] Geut H., Hepp D.H., Foncke E., Berendse H.W., Rozemuller J.M., Huitinga I., Van De Berg W.D.J. (2020). Neuropathological correlates of parkinsonian disorders in a large Dutch autopsy series. Acta Neuropathol. Commun..

[100] Jellinger K.A., Wenning G.K. (2021). Is Multiple System Atrophy a Prion-like Disorder?. Int. J. Mol. Sci..

[101] Ubhi K., Rockenstein E., Mante M., Inglis C., Adame A., Patrick C., Whitney K., Masliah E. (2010). Neurodegeneration in a Transgenic Mouse Model of Multiple System Atrophy Is Associated with Altered Expression of Oligodendroglial-Derived Neurotrophic Factors. J. Neurosci..

[102] Sacino A.N., Brooks M., Thomas M.A., McKinney A.B., Lee S., Regenhardt R.W., McGarvey N.H., Ayers J.I., Notterpek L., Borchelt D.R. (2014). Intramuscular injection of α-synuclein induces CNS α-synuclein pathology and a rapid-onset motor phenotype in transgenic mice. Proc. Natl. Acad. Sci. USA.

[103] Ferreira N., Richner M., van der Laan A., Bergholdt Jul Christiansen I., Vægter C.B., Nyengaard J.R., Halliday G.M., Weis J., Giasson B.I., Mackenzie I.R. (2021). Prodromal neuroinvasion of pathological α-synuclein in brainstem reticular nuclei and white matter lesions in a model of α-synucleinopathy. Brain Commun..

[104] Glanzer J.G., Enose Y., Wang T., Kadiu I., Gong N., Rozek W., Liu J., Schlautman J.D., Ciborowski P.S., Thomas M.P. (2007). Genomic and proteomic microglial profiling: Pathways for neuroprotective inflammatory responses following nerve fragment clearance and activation. J. Neurochem..

[105] Colonna M., Butovsky O. (2021). Microglia Function in the Central Nervous System During Health and Neurodegeneration. Annu. Rev. Immunol..

[106] Joers V., Tansey M., Mulas G., Carta A.R. (2018). Microglial phenotypes in Parkinson’s disease and animal models of the disease. Prog. Neurobiol..

[107] Heneka M.T., Golenbock D.T., Latz E. (2015). Innate immunity in Alzheimer’s disease. Nat. Immunol..

[108] Biber K., Neumann H., Inoue K., Boddeke H.W.G.M. (2007). Neuronal ‘ On ’ and ‘ Off ’ signals control microglia. Trends Neurosci..

[109] Mackenzie I.R.A. (2000). Activated microglia in dementia with Lewy bodies. Neurology.

[110] Ferreira S.A., Romero-ramos M. (2018). Microglia Response During Parkinson’s Disease: Alpha-Synuclein Intervention. Front. Cell Neurosci..

[111] Theodore S., Shuwen Cao B., McLean P.J., Standaert D. (2009). Targeted Overexpression of Human Alpha-Synuclein Triggers Microglial Activation and an Adaptive Immune Response in a Mouse Model of Parkinson Disease. J. Neuropathol. Exp. Neurol..

[112] Béraud D., Twomey M., Bloom B., Mittereder A., Ton V., Neitzke K., Chasovskikh S., Mhyre T.R., Maguire-Zeiss K.A. (2011). α-Synuclein Alters Toll-Like Receptor Expression. Front. Neurosci..

[113] Hou L., Bao X., Zang C., Yang H., Sun F., Che Y., Wu X., Li S., Zhang D., Wang Q. (2018). Integrin CD11b mediates α-synuclein-induced activation of NADPH oxidase through a Rho-dependent pathway. Redox Biol..

[114] Stefanova N., Reindl M., Neumann M., Kahle P.J., Poewe W., Wenning G.K. (2007). Microglial Activation Mediates Neurodegeneration Related to Oligodendroglial alpha -Synucleinopathy: Implications for Multiple System Atrophy. Mov. Disord..

[115] Park J., Paik S.R., Jou I.L.O., Park S.M. (2008). Microglial Phagocytosis Is Enhanced by Monomeric a -Synuclein, Not Aggregated a -Synuclein: Implications for Parkinson’s Disease. Glia.

[116] Zhang W., Wang T., Pei Z., Miller D.S., Wu X., Block M.L., Wilson B., Zhang W., Zhou Y., Hong J.-S. (2005). Aggregated -synuclein activates microglia: A process leading to disease progression in Parkinson’s disease. FASEB J..

[117] Sarkar S., Dammer E.B., Malovic E., Olsen A.L., Raza S.A., Gao T., Xiao H., Oliver D.L., Duong D., Joers V. (2020). Molecular Signatures of Neuroinflammation Induced by α Synuclein Aggregates in Microglial. Front. Immunol..

[118] Kim C., Ho D., Suk J., You S., Michael S., Kang J., Lee S.J., Masliah E., Hwang D., Lee H. (2014). Neuron-released oligomeric α-synuclein is an endogenous agonist of TLR2 for paracrine activation of microglia. Nat. Commun..

[119] Galatro T.F., Holtman I.R., Lerario A.M., Vainchtein I.D., Brouwer N., Sola P.R., Veras M.M., Pereira T.F., Leite R.E.P., Möller T. (2017). Transcriptomic analysis of purified human cortical microglia reveals age-associated changes. Nat. Neurosci..

[120] Zhang Y., Chen K., Sloan S.A., Bennett M.L., Scholze A.R., Keeffe S.O., Phatnani H.P., Guarnieri X.P., Caneda C., Ruderisch N. (2014). An RNA-Sequencing Transcriptome and Splicing Database of Glia, Neurons, and Vascular Cells of the Cerebral Cortex. J. Neurosci..

[121] Angelopoulou E., Paudel Y.N., Villa C., Shaikh M.F., Piperi C. (2020). Lymphocyte-activation gene 3 (LAG3) protein as a possible therapeutic target for Parkinson’s disease: Molecular mechanisms connecting neuroinflammation to α-synuclein spreading pathology. Biology.

[122] Aulić S., Masperone L., Narkiewicz J., Isopi E., Bistaffa E., Pastore B., De Cecco E., Scaini D., Zago P., Moda F. (2017). α-Synuclein Amyloids Hijack Prion Protein to Gain Cell Entry, Facilitate Cell-to-Cell Spreading and Block Prion Replication. Sci. Rep..

[123] Ferreira D.G., Temido-Ferreira M., Miranda H.V., Batalha V.L., Coelho J.E., Szegö É.M., Marques-Morgado I., Vaz S.H., Rhee J.S., Schmitz M. (2017). α-Synuclein interacts with PrP C to induce cognitive impairment through mGluR5 and NMDAR2B. Nat. Neurosci..

[124] Shrivastava A.N., Redeker V., Fritz N., Pieri L., Almeida L.G., Spolidoro M., Liebmann T., Bousset L., Renner M., Léna C. (2015). a-synuclein assemblies sequester neuronal a3-Na^+^/K^+^-ATPase and impair Na^+^ gradient Amulya. EMBO J..

[125] Choi Y.R., Cha S.H., Kang S.J., Kim J.B., Jou I., Park S.M. (2018). Prion-like Propagation of α-Synuclein Is Regulated by the FcγRIIB-SHP-1/2 Signaling Pathway in Neurons. Cell Rep..

[126] Laurén J., Gimbel D.A., Nygaard H.B., Gilbert J.W., Strittmatter S.M. (2009). Cellular prion protein mediates impairment of synaptic plasticity by amyloid-Β oligomers. Nature.

[127] Thacker B.E., Xu D., Lawrence R., Esko J.D. (2014). Heparan sulfate 3-O-sulfation: A rare modification in search of a function. Matrix Biol..

[128] Xu D., Esko J.D. (2014). Demystifying Heparan Sulfate—Protein Interactions. Annu. Rev. Biochem..

[129] Varki A., Cummings R.D., Esko J.D., Stanley P., Hart G.W., Aebi M., Darvill A.G., Kinoshita T., Packer N.H., Prestegard J.H. (2009). Essentials of Glycobiology.

[130] El-Hage N., Podhaizer E.M., Sturgill J., Hauser K.F. (2011). Toll-like receptor expression and activation in astroglia: Differential regulation by HIV-1 Tat, gp120, and morphine. Immunol. Investig..

[131] Fellner L., Irschick R., Schanda K., Reindl M., Klimaschewski L., Poewe W., Wenning G.K., Stefanova N. (2013). Toll-like receptor 4 is required for alpha-synuclein dependent activation of microglia and astroglia. Glia.

[132] Brudek T., Winge K., Agander T.K., Pakkenberg B. (2013). Screening of toll-like receptors expression in multiple system atrophy brains. Neurochem. Res..

[133] Letiembre M., Liu Y., Walter S., Hao W., Pfander T., Wrede A., Schulz-Schaeffer W., Fassbender K. (2009). Screening of innate immune receptors in neurodegenerative diseases: A similar pattern. Neurobiol. Aging.

[134] Rannikko E.H., Weber S.S., Kahle P.J. (2015). Exogenous alpha-synuclein induces toll-like receptor 4 dependent inflammatory responses in astrocytes. BMC Neurosci..

[135] Kim C., Kwon S., Iba M., Spencer B., Rockenstein E., Mante M., Adame A., Shin S.J., Fields J.A., Rissman R.A. (2021). Effects of innate immune receptor stimulation on extracellular α-synuclein uptake and degradation by brain resident cells. Exp. Mol. Med..

[136] Azarias G., Kruusmägi M., Connor S., Akkuratov E.E., Liu X.L., Lyons D., Brismar H., Broberger C., Aperia A. (2013). A specific and essential role for Na,K-ATPase α3 in neurons co-expressing α1 and α3. J. Biol. Chem..

[137] Bido S., Muggeo S., Massimino L., Marzi M.J., Giannelli S.G., Melacini E., Nannoni M., Gambarè D., Bellini E., Ordazzo G. (2021). Microglia-specific overexpression of α-synuclein leads to severe dopaminergic neurodegeneration by phagocytic exhaustion and oxidative toxicity. Nat. Commun..

